# Applying the RE-AIM framework to evaluate a holistic caregiver-centric hospital-to-home programme: a feasibility study on Carer Matters

**DOI:** 10.1186/s12913-022-08317-3

**Published:** 2022-07-19

**Authors:** Ee Yuee Chan, Ling Ting Wu, Emmalene Joo Yong Ng, George Frederick Glass, Robyn Hwee Teng Tan

**Affiliations:** 1 Nursing Research Unit, Tan Tock Seng Hospital, Singapore, Singapore; 2 Alice Lee Centre of Nursing Studies, Yong Lin School of Medicine, National University of Singapore, Singapore, Singapore; 3 Geriatric Education and Research Institute, Singapore, Singapore; 4 Singapore Social Service Research Centre, National University of Singapore, Singapore, Singapore

**Keywords:** Caregiving, Caregiver burden, Caregiver stress, Feasibility study, Implementation science, Person-centered care, Hospital

## Abstract

**Background:**

Prolonged caregiving of an older adult can cause family caregivers to be overwhelmed, potentially affecting the well-being of both the caregivers and their care-recipients. Carer Matters is a holistic hospital-to-home programme, centred on caregivers’ needs as their care-recipients transit from hospital to home. The programme was piloted to support caregivers through caregivers needs assessment, tailored resources, tele-support, training courses, and community support network. This study aimed to examine the feasibility of Carer Matters in a tertiary hospital in Singapore.

**Methods:**

This feasibility study was conducted on the pilot implementation from January to December 2021, during the Covid-19 pandemic. It adopted the Reach, Effectiveness, Adoption, Implementation, and Maintenance (RE-AIM) framework. The study highlighted quantitative data collected from key process indicators, such as number of caregivers screened, assessed on their needs and provided with assistance. Additionally, qualitative data was collected from in-depth interviews with 51 stakeholders involved in the implementation to examine their perspectives and experiences. These included family caregivers, clinician caregiver support nurses, hospital leaders and community partners.

**Results:**

During the pilot, 550 caregivers were enrolled. All caregivers received educational resources when they completed the needs assessment, while 69 of them who reported high burden were given tele-support and 252 attended our caregiver training courses. Despite initial recruitment challenges and obstacles to adoption, stakeholders interviewed found Carer Matters to be effective in providing caregivers with emotional support, knowledge and skills that improved their caregiving abilities, and reduced their sense of isolation and caregiving stress. Among caregivers, the training courses were effective with majority of caregivers agreeing that the courses addressed their needs (99%) and improved their knowledge of the relevant disease conditions (97%). Programme maintenance considered among stakeholders included strategies such as multipronged approach in recruiting caregivers and inviting caregiver advocates to share their experiences.

**Conclusion:**

This feasibility study highlights that Carer Matters is a valuable component to the ecosystem of support for family caregivers and their care recipients. Carer Matters extends the current patient-centric care model to a more holistic post-discharge continuity of care for both caregivers and their care-recipients, improving and maintaining their overall well-being to better allow transition from hospital-to home.

**Trial registration:**

Feasibility Study of Project Carer Matters for Family Caregivers of Persons with Dementia (NCT number: NCT05205135).

**Supplementary Information:**

The online version contains supplementary material available at 10.1186/s12913-022-08317-3.

## Background

An exponentially ageing population and increasing concomitant chronic diseases have ushered a normalcy of older adults being cared for at home [1], ageing in place instead of being institutionalized. Their activities of daily living and other needs are often managed by their family members, who often take on the role and responsibilities of an informal caregiver, in addition to their other social and familial responsibilities. However, the fulfilment of these responsibilities without meaningful respite or relief for prolonged periods can cause significant caregiver stress, and build up into caregiver burden [2]. This burden is often associated with negative impacts on caregivers’ emotional, physical, and psycho-social well-being and financial capability [3, 4]. Often entering the role without sufficient preparation and support such as skills training and self-care knowledge, caregivers might not be able to deliver an optimal quality of care due to the high levels of burden they experience [3, 5]. This problem is especially salient during their care recipients’ transition from hospital to home as caregivers are met with an onset of new responsibilities in assisting the continued recovery and support of their loved ones. Hence, healthcare providers should utilize this opportune period to equip and support caregivers [6].

Caregiver burden is a prevalent issue in Singapore, a rapidly ageing nation which will have 900,000 individuals aged 65 and above by 2030 cared for by a shrinking pool of family members [7]. Local studies have demonstrated the effects of unresolved caregiving stress on the healthcare system. This includes increased emergency room attendance and prolonged hospital stay of the care-recipient [8]. Furthermore, caregivers tend to devote most of their time to caregiving and learning to better manage their care-recipient’s condition, instead of their own well-being and self-care [9]. This may eventually result in caregivers developing adverse health conditions such as chronic diseases and depression [10].

The transition from hospital-to-home is a recognized critical opportunity to promote patient safety and high-quality care for older adults and their caregivers [11]. Key mechanisms to equip such skills and knowledge include services such as care coordination and patient education [12]. Consequently, patient discharge outcomes such as readmission rates will improve, emphasizing the need for healthcare providers to offer those services [12]. However, transitional care support for older adults remains specifically focused on the needs of the patients [11]. This could ignore the essential role that caregivers play, and have potential repercussions on the recovery of the older adults.

Our earlier research on the profiles of caregivers highlighted that caregivers have complex and diverse needs [13]. This necessitates a multifactorial framework to consider the needs of caregivers, beginning with the identification of caregivers in need and following up with targeted support. Support can be delivered through holistic interventions to improve caregivers’ coping abilities and prevent institutional placement of their care-recipients [14]. The identification of caregiver stress can be performed using specially-designed tools [15]. An opportune period for such screening is during the hospitalization of the older adults [15, 16].

Similar studies have also revealed and emphasized the importance of maintaining a continuity of care to alleviate the stress that caregivers experience during the post discharge period of their loved ones [3, 16–18]. Notably, the need for healthcare providers to recognize caregiver burden and intervene in a timely manner has been reiterated in many of those studies [16, 17]. Local studies exploring better caregiver support as part of the patient discharge experience recommend the use of an assessment tool to identify caregiver stress as a stepping-stone towards providing holistic and tailored support to caregivers [3, 16].

An effective and sustainable hospital-to-home programme is envisioned to streamline the system of support for caregivers, equipping them with the necessary care management skills and knowledge, and provide them with emotional support needed to sustain long-term caregiving [19]. Building on the established model of a holistic ‘hospital-to-home’ care for patients, we aimed to examine the feasibility of such a  programme for caregivers [16, 17]. By addressing needs that surface from the inpatient setting to the home, we can establish a support framework to help caregivers and their loved ones thrive at home, within their community [16, 17].

In this spirit, we piloted Carer Matters, a holistic hospital-to-home programme for family caregivers of hospitalized patients at a tertiary hospital in Singapore. This programme applies a multi-pronged strategy to support, empower and equip caregivers with the knowledge, skills and confidence to better manage the care of their loved one. This was delivered through a variety of media such as telesupport, self-directed learning materials, training courses, and linking caregivers to community support networks.

While this programme was originally designed for caregivers of persons with dementia, we decided to expand the participant eligibility criteria to include all caregivers of older adults aged 65 years and above due to similarities in their needs.

To our knowledge, Carer Matters is one of the first caregiver-focused programmes available to holistically support caregivers in their transition from hospital to home. Hence, a thorough evaluation was required to ascertain the utility and potential of such ‘care for caregivers’ before assessing if it was suitable for upscaling. The assessment of the feasibility of a programme such as Carer Matters would enable us to improve the process of “identifying, screening, equipping, engaging, and supporting framework” caregivers in need during the immediate post discharge period [19].

In this study, we examined the feasibility of implementing Carer Matters in the hospital-to-home setting. The implementation of Carer Matters coincided with the outbreak of COVID-19, decreasing caregivers’ access to many resources and services due to social distancing measures. As such, Carer Matters had to adapt to the changing hospital policies and protocols in response to the evolving COVID-19 situation.

## The study

### Design

A multi-method feasibility study was conducted [19]. The protocol for this study was registered in ClinicalTrials.gov (NCT05205135) [18]. Due to the complexity of the program with multiple components, both quantitative and qualitative data from various sources were collected. Qualitative data were collected from both caregivers who participated in the programme as well as key stakeholders involved in implementing it. By interviewing different groups of stakeholders, we examined the programme’s feasibility from multiple perspectives, allowing triangulation of data [20]. Simultaneously, quantitative process indicators were also included to examine the extent of the programme’s outreach. Due to the reduced number of eligible caregivers in the pilot wards over the study period due to the COVID-19 pandemic restrictions, quantitative data on caregiver outcomes were only captured from participants of our training courses.

#### RE-AIM framework

The Reach, Effectiveness, Adoption, Implementation and Maintenance (RE-AIM) model is a comprehensive framework that offers a structured approach to evaluate public health interventions [21]. We employed RE-AIM as a guide for the development of interview questions, data collection, organization, and analysis of our study, to aid us in understanding the efficacy, feasibility and scalability of Carer Matters, as well as the challenges in its implementation [22]. A deductive thematic analysis approach was adopted to code and identify themes within the transcripts. The five components of RE-AIM were contextualized to the study as shown in Table 1.

**Table 1:** RE-AIM Evaluation of Carer Matters (Adapted from Holtrop et al. (Adapted from Holtrop et al. [23])

Definition of RE-AIM	In the Context of Carer Matters (CM)	Interview Group
**Reach** is the absolute number, proportion, and representativeness of individuals who are willing to participate in a given intervention or programme [23]	What factors contributed to caregivers joining or not joining CM? What might have been done to motivate more caregivers to participate?	Family caregivers (CG) Ward nurses (WN) Ward champions (WC) Caregiver support nurses (CS) Community-based clinicians (CC)
**Effectiveness** is the impact of an intervention on outcomes, including potential negative effects, quality of life, and economic outcomes [23]	To what extent did the intervention contribute to the intended outcomes? What other factors contributed to the results? Were the results meaningful in aiding caregivers through emotional support, medical advice and caregiver trainings?	Family caregivers (CG) Caregiver support nurses (CS) Hospital leaders (HL)
**Adoption** is the absolute number, proportion, and representativeness of settings and the individuals within those settings who deliver the programme and are willing to initiate a programme. Use of qualitative data to understand setting level adoption and staff participation [23]	What were the motivating factors and barriers to adopting the intervention? To what extent was the intervention adopted?	Ward nurses (WN) Ward champions (WC) Hospital leaders (HL) Community-based clinicians (CC)
**Implementation** is the fidelity to programme protocol and adaptation made to invention during study. Costs of intervention in time and money. Consistency of the implementation across staff, time, setting, and subgroups — focus is on process [23]	By whom, how, and when was CM implemented? What were the motivating factors and barriers to implementing the intervention? How did implementation affect outcomes? How and why was the intervention adapted or modified over time?	Ward nurses (WN) Ward champions (WC) Caregiver support nurses (CS) Community partners (CP)
**Maintenance** is the extent to which a programme becomes institutionalized or part of the routine of organisational practices and policies. If and how the programme was adapted long-term [23]	What are the considerations to continue (or discontinue) the intervention after the pilot phase? What will/should be sustained, what will be discontinued, and what will be modified? And why?	Hospital leaders (HL) Community-based clinicians (CC)

### Carer Matters programme

Carer Matters was piloted in a 1,700-bedded tertiary hospital in Singapore, for a duration of 12 months from January 2021. Six wards, consisting of four acute wards and two sub-acute wards with a total of 236 beds, were involved. The wards were chosen for their high volume of admitted older adults.

**Fig. 1:**
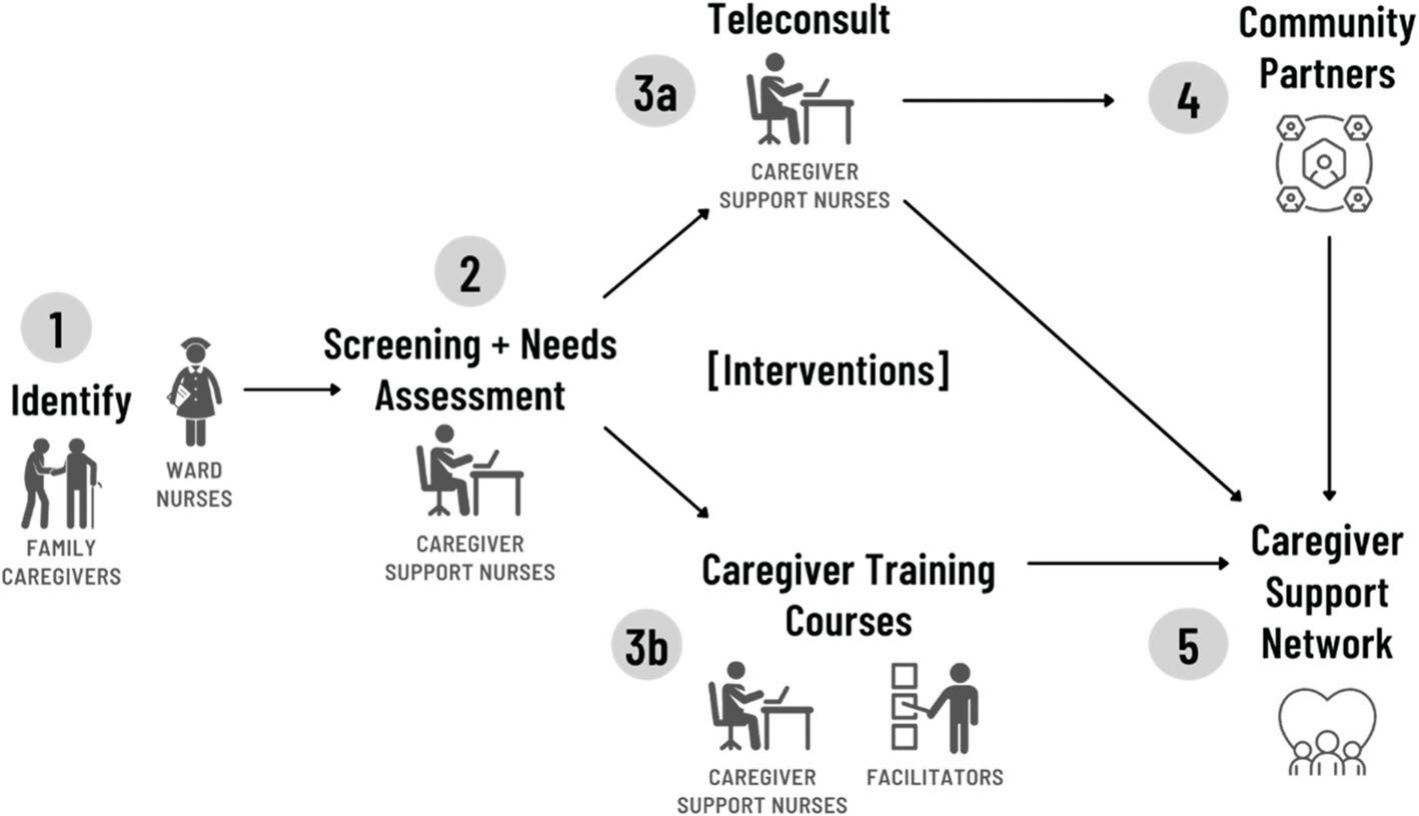
Carer Matters Flowchart

The programme’s activities are delivered by caregiver support nurses —experienced registered nurses with geriatric backgrounds and trained to address caregiver enquires and needs in managing the care of their loved ones and caregiver self-care. The programme consisted of five components (see Fig. 1). Component 1 comprised of the identification of eligible family caregivers of hospitalized older adults aged 65 and above. The identification was conducted by trained inpatient ward nurses. Component 2 comprised of the enrolment of caregivers upon their completion of a needs assessment form. These caregivers were subsequently provided with a tailor-made resource package. A 9-item Zarit Burden Interview was included in the needs assessment to identify caregivers at-risk of high burden and stress (scoring ≥ 17 out of 36) [19]. Component 3a involved the provision of tele-support by caregiver support nurses to caregivers identified to be at-risk of burden and stress. Component 3b involved the delivery of caregiver training courses to enhance caregiving knowledge and skills. Component 4 entailed the building of pipelines of access from the hospital to community partners who provide services for patients such as day care, respite care and case management. Finally component 5 connected caregivers in need to a support network of community service providers and peer caregivers so that they will have continual support in their caregiving journey. Hence, the programme provided caregivers with a continuum of support as they sequentially engage in the abovementioned components from hospital to home. A detailed description of the programme and its components was reported in our study protocol [18].

The eligibility criteria for caregivers to participate in Carer Matters were: family caregivers of an older adult aged 65 years and above who were (1) 21 years of age and above, (2) caring for family member/s receiving home-based care, and (3) able to read and converse in English or Mandarin.

To effectively rollout the pilot programme across the wards, caregiver support nurses trained and supported ward champions — nurses from inpatient wards who functioned as representatives for the programme — who would guide their colleagues on the identification and enrolment of caregivers. Caregiver support nurses also partnered community-facing clinicians, medical social workers, and occupational therapists to streamline the flow of post-discharge support that segues into the community. In addition, key community partners were identified as case management liaisons by caregiver support nurses for caregivers who required further support in the community. Hospital leaders also provided insight and guidance on the delivery of the programme for integration with the hospital’s strategic development initiatives.

### Sample/participants

#### Quantitative data

Convenience sampling was applied, collecting data from all eligible caregivers who received the Carer Matters’ interventions, including at least one of the training courses offered over the pilot period from January to December 2021.

#### Qualitative data

Stratified purposive sampling was used to recruit study participants who were directly or indirectly involved in the programme’s implementation. Stratification was used to include both caregivers and healthcare providers involved in different components of Carer Matters to elicit in-depth evaluation according to the RE-AIM categories (refer to Table 1). Participants included family caregivers recruited into the programme (*N* = 27), ward nurses and ward champions (*N* = 9), caregiver support nurses (*N* = 6), community-based clinicians such as medical social workers and nurses (*N* = 5), members from community organisations that partnered the programme (*N* = 2) and hospital leaders (*N* = 2). Potential interview participants were screened for eligibility based on the following criteria: (1) above the age of 21 years old, (2) able to converse in either English or Mandarin, and (3) willing to have their interviews audio recorded.

### Data collection

Semi-structured, individual interviews were conducted with the key stakeholders of Carer Matters. Using the Reach, Effectiveness, Adoption, Implementation, and Maintenance (RE-AIM) framework as a guide, the interview guide (see Supplementary Materials) focused on examining participants’ views on the programme, barriers to adoption and implementation, and sustainability issues. One researcher who was well-trained in qualitative research methods and independent of the development and implementation of Carer Matters conducted all the interviews. While we initially planned for all our interviews to be conducted face-to-face, physical and social distancing measures were mandated hospital-wide in May 2021 to reduce the transmission of COVID-19. In response, we transitioned to conducting interviews over the online platform Zoom. All interviews were audio-recorded and lasted approximately 30 to 60 min.

Quantitative data were collected from participant logs to obtain key process indicators such as (1) the number of caregivers screened, (2) the number of family caregivers assessed on their needs and (3) the number of family caregivers provided with assistance. Also, pre- and post-surveys were administered to participants of the caregiver training courses. Dementia knowledge was assessed using an 8-item questionnaire designed by three expert dementia nurses, assessing knowledge on the nature of dementia and how caregivers can manage behavioural and psychological symptoms of the disease. Participants’ scores were summed up with a potential range of 0 to 8, with higher scores representing better knowledge. Other questionnaires captured psychological indicators such as the use of task-focused or emotion-focused coping, caregiver burden, caregiver anxiety and depression, caregiver competence, and caregiver mastery; utilising the Coping Inventory for Stressful Situations, a modified version of the Zarit Burden Inventory [24], the Hospital Anxiety and Depression scale [25], the Caregiver Competence scale [26], and the Personal Mastery scale [27] respectively. The items were positively scored, with lower scores reflecting lower burden anxiety, depression, competence and mastery accordingly.

### Data analysis

Thematic analysis was conducted according to the theoretical framework RE-AIM [28]. Verbal transcripts were first categorized by the different stakeholder groups. Two research members were given time to read and re-read the transcripts to ensure that they were immersed into the data. They then independently coded each verbatim transcript. Codes were categorised and grouped into sub-themes according to the five components under the RE-AIM model. Disagreements were settled through discussion with research members. Finally, the research team reviewed and compared codes from all stakeholder groups to identify a coherent narrative.

Quantitative data analysis was conducted using Jamovi version 1.6.23 [29]. Descriptive statistics were used to describe caregiver-specific outcomes captured from participant logs and pre- and post-training courses surveys. We examined participant outcomes from two of our programmes, the Understanding Dementia course and our Coaching, Advocacy, Respite, Education, Relationship, Simulation (CARERS) programme. Parametric tests were applied to compare changes in dementia knowledge captured in the Understanding Dementia course chosen as it was the most-subscribed programme by caregivers. However, due to the small number of participants for the intensive 8 weeks CARERS programme, non-parametric tests were used to compare the change in median for coping, burden, anxiety, depression, competence and mastery scores before and after the courses [30]. Statistical significance was set as *p* < 0.05.

### Rigour and validity

Our qualitative analysis complied with the recommended principles for qualitative analysis when applying the RE-AIM framework [23]. Strategies to enhance the credibility, dependability, confirmability and transferability of the data were adopted to establish trustworthiness [31]. The research questions were carefully developed and guided by a validated framework (RE-AIM). One experienced qualitative researcher who was not involved in the conceptualization and implementation of Carer Matters, conducted all the interviews to ensure credibility of the data collection. The coding process and identification of any new concepts were discussed and verified by the team to ensure correct and consistent interpretation throughout the analysis. To ensure confirmability, the team discussed any differences in perspectives and came to a consensus on the themes and subthemes. Field notes were also taken during the interview and checked back with the data. Transferability of data was also enhanced through purposive sampling to include a diverse group of key stakeholders for Carer Matters. Taken together, these measures helped ensure internal and external validity, reliability and objectivity of our findings [31].

The questionnaires used in this study include the Caregiver Competency Scale, the Caregiver Mastery Scale, the Hospital Anxiety and Depression Scale and the Zarit Burden Interview. These scales have been well validated and applied in previous studies of family caregivers [3, 15, 32].

## Findings

### Demographic data of interview participants

Table 2 depicts the demographic data of the 51 participants recruited for individual interviews, of which there were 27 caregivers and 24 healthcare providers – ward nurses, clinical staff, community partners, and hospital leaders. Caregivers were mostly of middle age (mean 53 ± 13.4 years old), female (74.1%), Chinese (92.6%), and children of care-recipients (74.1%). Most caregivers were living with their care-recipients (77.8%), about half lived in 3 to 4 room flats (55.6%), and about half were employed full-time (51.9%). The majority of caregivers reported that they had received no financial assistance (63.0%). Healthcare providers interviewed were mostly female (95.8%) and Chinese (58.3%). They had an average of 12 years of care experience in the field of geriatric-related care.

**Table 2:** Socio-demographic characteristics of interview participants

Demographics	*Caregivers N (%)/ Mean* ± *SD*	*Healthcare providers* *N (%)/ Mean* ± *SD*
**Age (years)**	53.8 ± 13.4	36.1 ± 7.09
**Gender**		
Male	7 (25.9%)	1 (4.2%)
Female	20 (74.1%)	23 (95.8%)
**Relationship to care-recipient**		
Spouse	4 (14.8%)	
Child	20 (74.1%)	
Others	3 (11.1%)	
**Living with care-recipient**	21 (77.8%)	
**Ethnicity**		
Chinese	25 (92.6%)	14 (58.3%)
Malay	0	3 (12.5%)
Indian	0	2 (8.3%)
Others	2 (7.4%)	5 (20.8%)
**Marital status**		
Single	10 (37.1%)	
Married	13 (48.1%)	
Divorced/Separated	2 (7.4%)	
Widowed	2 (7.4%)	
**Highest education level**		
Secondary	11 (40.7%)	
Post-Secondary	5 (18.6%)	
Degree and above	11 (40.7%)	
**Working status**		
Full-time	14 (51.9%)	
Part-time	5 (18.5%)	
Unemployed	2 (7.4%)	
Retired	6 (22.2%)	
**Housing type**		
1–2 room flat	1 (3.7%)	
3–4 room flat	15 (55.6%)	
5 room flat /executive apartment	4 (14.8%)	
Condominium/Landed property	6 (22.2%)	
Others	1 (3.7%)	
**Form of financial assistance**		
None	17 (63.0%)	
Family	4 (14.8%)	
Government	6 (22.2%)	
**Form of help in caregiving**		
None	7 (25.9%)	
Paid helper	12 (44.4%)	
Family members	10 (37.0%)	
Others	1 (3.7%)	
**Years of care experience in older person-related field**		12.3 ± 6.9

### Reach

Under “reach” we discuss the primary mode of recruitment, that is, the ward nurses’ engagement with caregivers at the inpatient setting during visiting hours as well potential factors that could have affected programme recruitment. Ward nurses introduced Carer Matters to identified caregivers and invited them to enrol for the programme by completing the needs assessment. The needs assessment accessed through scanning a QR code that would direct caregivers to an electronic form.

### Recruitment of caregivers onto Carer Matters

During the study period, a total of 550 caregivers enrolled onto Carer Matters by completing the caregiver needs assessment. All of them received relevant educational resources based on their caregiving needs. Among these, 227 caregivers were assessed to be at-risk of caregiver burden based on their Zarit burden assessment scores. 157 of the 227 (69.2%) managed to be reached by the caregiver support nurses and were further assessed on their stress levels. 69 of the 157 (43.9%) were further supported through tele-support services. One caregiver declined further contact. Including participants of the caregiver seminars organized by Carer Matters, there were a total of 252 caregivers who attended caregiver training courses.

Our dropout rate was 1% (7 out of 550), consisting of caregivers who did not complete the training course they signed up for. While these caregivers did not complete their training course, they received the educational resources mapped to their needs. That way, they would still receive information to help in their caregiving. Further insights were elicited in the interview data below.

Most caregivers completed the needs assessment without hesitation, as they understood that the programme was an initiative by the hospital, trusting its reputable source and the healthcare providers.

“If it’s in a hospital context, we [caregivers] are not so worried. But if it’s on the streets, I’ll most probably say, ‘Sorry, I don’t even take surveys.’" (CG8)

Most caregivers, like CG3, also found the questions in the needs assessment quite “straightforward (and) simple” to complete.

As the programme was new, some caregivers questioned the purpose of the needs assessment as they “could not understand the importance [and]…benefits of (it)” (WN2). Others did not identify themselves as a caregiver of the patient as they associated caregiving responsibilities to the tasks performed by their hired domestic helper.

“Caregivers told us, 'I am not the caregiver, I have a helper.'” (WN3)

Some caregivers did not feel the need to participate in Carer Matters as they prioritized care for their care recipient instead of for themselves.

“…you tell me maybe [the caregiving-related programme] is at night, but I doubt [that I can join], because my mum is still in the hospital, I still have to come down [to the hospital], I have to visit her. It is very difficult for me [to attend the programme as well]. (CG11)”

Many caregivers felt that joining Carer Matters would be unnecessary, and that caregiving was their duty to do so in their individual capacity. However, caregivers were more amenable to join able after learning from the ward nurses how the programme could benefit themselves.

“…once they see [how the programme helps them], then they will happily [sign up].” (WN3)

### Factors that could have affected caregiver recruitment

While the decision to participate in the programme was voluntary on the caregivers’ part, we identified several factors that might have influenced the take-up of Carer Matters. The introduction of the programme was dependent on the nurse being physically present with the caregiver to explain the programme. Hence, if the nurse was unavailable, it was much harder to identify caregivers and inform them of the programme. This was contributed by visiting hours coinciding with peak ward activities conducted by the nurses like “feeding, turning and giving medications” (WN1), thereby reducing the opportunity in nurse-caregiver interactions for a detailed introduction and explanation of Carer Matters to the caregivers.

“…when the caregivers come, it is usually during the visiting hours, then…the nurses may be busy…” (WN9)

Moreover, when caregivers visit, the restricted time spent with their loved ones were precious to them, leading them to decline or delay filling up the needs assessment. Community partners additionally shared that from their experience, caregivers were often unaware of their needs unless they were deep in crisis, causing many to be insufficiently prepared for potential challenges. As a result, many might not have had reached out and potentially benefitted from the programme until they were facing crisis.

“Usually when a caregiver starts talking about needing help, it’s because they are desperate for help. Caring for somebody, managing it you know, I’m doing fine, you know until one day, I just [burn] out…So it’s a lot of crisis management, I would think.” (CP1)

However, while caregivers initially reported that it was hard to find time for the training courses offered due to their pre-occupation with caregiving and work, they were more willing to join Carer Matters when ward nurses approached them with the appeal of them being able to help other caregivers through the programme.

“It’s a project not just to help you ([the caregiver]) but to help other caregivers as well. We are also finding out how to help other caregivers in the future,” that kind of thing, then they will be more keen (to join). (WC2)

"…because [the programme] will benefit (the) whole family, empower the patients, caregivers and all. It will help." (WN5)

Sufficient time to explain the intrinsic benefits of Project Carer Matters to caregivers was therefore a crucial factor in encouraging caregivers’ participation in the programme.

“You need some time to speak to them, explain to them [what is] [Carer Matters], [which is] to lift their burden, to give ...some help from the community. Actually, they are more okay." (WN4)

### Effectiveness

Under ‘effectiveness’ we assessed how caregivers benefitted from each intervention of Carer Matters—the programme’s educational resources, telesupport by caregiver support nurses and caregiver training courses to equip them with knowledge and skills. We also identified the areas for improvement in each intervention.

### Educational resources (Component 2)

Generally, caregivers found the educational resources, such as information on their care-recipient’s condition, accessible and useful. For most first-time caregivers in particular, like CG12 who might not know where to seek help, a list of collated emergency contacts and links to community services such as respite care, counselling, and support groups was especially "useful" and "helpful" as it prevented information "overload" and assisted them in “hunt(ing) for [the right contact information]”.

“I do gain from…when they send us links to support…home care or some nursing home,…the list of places where we can go for help. Yeah. Those are actually quite useful to us.” (CG18)

The above was concurred by many nurses like WN2, who feel that Carer Matters provides a “very good platform” in assisting first-time caregivers who “do not know how to reach out” for the relevant support and resources “in the community” that can help alleviate their caregiving burden*.*

“Obviously, this programme will definitely benefit the caregivers…” (WN7)

In addition, the resources sent through email made it convenient for caregivers to access them. However, caregivers with lower digital literacy and “who are not very good in technology” “faced problems” (WN6), and found the electronic resources lengthy and less effective. Those were often older caregivers who shared that they preferred resources in the form of instructional videos or audio-visual aids; “stuff that [they] can just log on and listen to”. (CG15). This points towards a need for printed or alternate forms of resources so less digitally-literate caregivers can still access and utilize them.

### Tele-support (Component 3a)

During the study period, tele-support was offered to 69 caregivers identified to be at-risk of caregiving stress. Caregivers who were followed up through tele-support can reach the caregiver support nurses through the helpline any time within their working hours. During the tele-support sessions, caregivers “appreciated” that “[the nurses] tried to update” (CG3) them via calls, who also provided them with much-needed medical advice and emotional support, as well as a platform to “ventilate” their emotions and “feel better” afterwards (WN7).

“I’m able to express better and I get replies from the experts [caregiver support nurses] straight away. Now I get some emotional support from them, I feel quite relieved in the sense that I let out whatever my problems, and they [caregiver support nurses] are able to listen and understand me, and sometimes give me some advice pertaining to that specific problem.” (CG18)

Nurses who were interviewed valued the emotional support provided via by Carer Matters. A ward nurse shared: “For the carer, I think that emotional support is quite important.” (WN1).

Through tele-support, the caregiver support nurses also referred caregivers to relevant community resources according to their needs. Many ward nurses like WN7 felt that such ‘support’ increases caregivers’ coping ability and reduces caregiver ‘burnt out’ and in turn, the number of care-recipients sent to nursing homes as it “increases [caregivers’] confidence [in] bring[ing] their loved ones…home”; this better preserves care-recipients’ “dignity”, enabling them to age-in-place in “the community” (WN9).

“… then if you can send them for a [Carer Matters] programme, you will…link [the caregivers] with those community support [for respite care]…so they have more time for themselves. Because …caring for the sick patients itself,…can be stressful for the caregivers; they do not have time…to care for themselves. So if at least there are some community partners coming in to support them, it will be helpful for them. At least they are not alone.” (WN8)

The nurses who were interviewed also shared that lowered caregiver stress via community support can potentially help to reduce care-recipients’ hospital re-admission rates.

“Having carers’ stress is also…not a good thing to have…having community support to help [the caregivers], …they will feel more well-supported in our community…[then] at least will not have…more frequent [hospital] admissions…So it is good for them to get some support.” (WN9)

Additionally, community partners shared that the direct connection from hospital to community accelerated administrative processes, ensuring that caregivers received prompt support.

“While trying to help the caregiver that she [caregiver support nurse] referred, I [community partner] needed information from the hospital…I needed the doctor’s memo and the hospitalisation record, so she [caregiver support nurse] managed to get it together… Without her help, I would have to wait for the next appointment for the [caregiver] to speak to the doctor.” (CP2)

At times, the caregiver support nurses even went the extra mile to support caregivers, helping to arrange clinic appointments or engaging clinicians for documents. One caregiver shared that the caregiver support nurse who assisted her was “very helpful, she applied [for] [an initiative/fund]…so that part, she is helping me to manage.” (CG3).

Such assistance was greatly appreciated by caregivers interviewed, with some perceiving Carer Matters as a “very great initiative” as it made them feel less “deserted” and a platform where “staff [can] teach [caregivers] what to do” when the latter face issues (CG12). This support was especially so and “benefi[cial]” to caregivers who were solely managing care by themselves, with “no helper [and who] were …caring for [their care-recipients]” (WN8). Another ward nurse shared that it is important to “give the caregiver enough support, to let them know [that] someone is behind [them]… and if they [are] facing any difficulties, any stress, they will [have] someone to talk to, to rely on…(WN7).

While the tele-support service was greatly appreciated, the availability of support was limited by the caregiver support nurses only being able to provide assistance during office hours – Monday to Friday, 8am-6 pm.

### Caregiver training courses (Component 3b)

A series of courses (Table 3) were developed and offered to caregivers according to their needs, such as to gain a better understanding of their care-recipients’ medical condition and to improve their caregiving skills and mastery. The majority of caregivers who attended the courses were newer caregivers with less than three years of experience (54%), aged more than 50 years old (63%) with an undergraduate degree (54%) and working full-time (57%) while caring for their parents (79%).

**Table 3:** List of caregiver training programmes developed [33]

Training Programmes	Programme Description
CARERS (Coaching, Advocacy, Respite, Education, Relationship, Simulation) Programme [34]	A therapeutic group intervention that features a unique hands-on simulation exercise, in the presence of a standardized patient, to practice the application of problem-solving techniques. The programme is co-led by two facilitators and held on a weekly basis over the course of eight weeks in small groups of four to six participants
How To Care At Home Programme	An interactive group programme that aims to build caregiving skills and provide emotional support for family caregivers. Sessions are tailored to central themes of caregiving (changing relationship, community resource navigation, future planning, self-care)
Understanding Dementia	This programme helps caregivers understand dementia, the nature of Behavioural and Psychological Symptoms of Dementia (BPSD) and general approaches to challenging behaviours
Problem-solving techniques	This programme introduces a five-step problem solving technique adapted from the CARERS programme. This is a group session tailored to help caregivers address practical problems faced [34]
Self-care techniques	This programme will help caregivers recognize the importance of self-care and learn practical self-care tips
Caregiving Essentials	This group programme will help caregivers understand more about caregiving and provide practical caregiving tips
Public forums/Seminars	Caregiver Seminar 2021

Generally, caregivers acknowledged the usefulness of the extensive information provided during the courses, a perspective similarly shared by the nurses. “It is because they really see the value that this…education, …the workshops done [for] them … help them.” (WN6).

Interestingly, there were differing preferences on the inclusion of caregivers with care-recipients at different stages of their illness in the same course. On one hand, some caregivers preferred having courses catered to caregivers with loved ones at specific stages of illness.

“Our parents are at different stages of dementia, so each of us actually have our own challenges…, because in my current situation, I have my other challenges that are very different from someone who is in the beginning stage.” (CG1)

On the other hand, the caregiver support nurses who delivered these training courses felt that having caregivers with loved ones at different stages of illness attending the same course has its benefits such as encouraging social learning and support from their universal circumstances.

“These caregivers are caring for people with dementia in different stages, but I think that’s the beauty of it. For example, if a caregiver is sharing about the care-recipient who’s [in] the early stages, the caregivers in the late stages are able to provide some of their experiences in how they actually dealt with it.” (CS5)

This insight was validated by many caregivers like CG16 who expressed that the Carer Matters program “is very, very good”, as they took delight in being able to learn from fellow caregivers on how to “manage and cope” with their respective caregiving situations, in which the mutual sharing of “eye-opening” (CG15) caregiving learning experiences. This was greatly valued and appreciated by the caregiver participants as it “empower[ed]" them (WN5) and simultaneously provided them ‘a voice…to actually share how they feel” (WN7) with other caregivers.

Moreover, knowing that someone else is going through “the same journey” additionally provided them with a sense of “feel-good” (CG21) camaraderie which alleviated their isolation.

“What’s important for all the caregivers is that when you start to hear the story of other people, you know that you’re not alone. It helps, you know?… a support group that they know who to talk to. Sometimes you may not have [a] solution, but just talking to somebody, someone listening to you, I think it also helps.” (CG12)

“A ward nurse shared that equipping caregivers through targeted courses helps, “because we are not only thinking about the patient here, but we also think about [the caregiver]…if you [caregiver] think that someone is caring for you [caregiver], understand how you have been struggling, I think it helps. It is helpful for them [caregivers].” (WN7)

Moreover, such courses enabled nurses like WN8 to “understand better how [caregivers] are stressing” and “their point of view on taking care of their…loved ones” so that they understand better “how [and] what else [they] can [do to] help” the caregivers cope with their caregiving burden*.*

Sixty-one caregivers attended the Understanding Dementia course and rated the training positively, with the vast majority agreeing that the course addressed their needs (99%), improved their knowledge of the relevant disease condition (97%) and would recommend the course to others (97%). The course was found to be effective in improving caregiver’s knowledge on dementia (*N* = 61) with an improved score of dementia knowledge from 5.39 (SD = 1.63) to 6.81 (SD = 1.32) after participating in the course (*p* < 0.001), a mean increase of 1.41 points (95%CI: 0.87–1.96).

The effectiveness of the various training courses such as Understanding Dementia was acknowledged by many nurses as it helps caregivers better understand and “know the behaviour of their [care-recipients]”, they can “at least manage” (WN9) their loved ones better, and also “increase their coping ability [and] caregiving mastery…” (WN5).

“…with better knowledge and by understanding the [care-recipient’s] conditions…I think it will benefit the caregivers…because this is not just about the patient, it is also [about] how you can help…how to ease [caregiver]…stress…” (WN8)

Improvement in the CARERS course was less notable due to the small number of caregivers (*N* = 16) who could attend the intensive eight-week course – changes in caregiver-reported outcomes of burden, anxiety and depression, caregiver mastery and competence were not statistically significant. However, the qualitative feedback shared above demonstrated its benefits to caregivers who attended.

The training courses’ intended outcomes may have been affected by the shift from face-to-face sessions to online sessions due to the COVID-19 situation. For instance, a component of the CARERS training course includes a simulation activity where caregivers learn through role-play and re-enactment of caregiving scenarios at home. Some caregivers revealed that it was challenging to role-play over the Zoom platform.

“It [CARERS training] lacks… physical interactions. Because through Zoom, it’s a new thing right now, so we are all trying to adapt, but if it’s [conducted] face-to-face, I think it will be much better, especially for the simulation part.” (CG26)

To overcome the technological barrier, caregiver support nurses adapted by being more precise when explaining the process and expected outcomes of the simulation, instead of merely providing instructions on the procedure:

“We [caregiver support nurses] let them know what the expected outcomes are, I think this is quite important. We will let them know usually the caregivers who are doing simulation, they need to be exact with the tones, the expressions, and everything”. (CS6)

This enabled caregivers to gain a better understanding of what they should expect and how they should role-play to acquire the intended benefits of the exercise.

### Adoption

Under ‘adoption’ we focused on the role of nurses in the pilot wards and the role community partners played in helping to support caregivers in the community; alongside the facilitators and barriers of their involvement.

### Adoption by nurses in piloting wards and facilitators/barriers

Carer Matters was implemented amidst the COVID-19 pandemic. The rapidly changing protocols and policies brought about by the evolving COVID-19 situation could have impeded ward nurses’ ability to adopt the programme into their routine work. Citing an example of restricted visiting hours, a hospital leader shared an observation:

“In this climate [visitation restriction due to COVID-19], most of the time when they [nurses] talk to caregivers, caregivers scold them because caregivers [prefer to focus on their loved ones]. The caregiver visits only for 30 min, so the caregivers face a lot of stress and frustration…” (HL2)

Nurses highlighted that the COVID-19 situation had resulted in more hospital admissions, leading to an increased workload in terms of managing more patients in the wards. In addition, nurses highlighted that they are too engrossed in their care for their patients and the daily routines.

“…Sometimes when I go into the ward, I am already immersed into the ward. I forget about everything else [including Carer Matters] that I am supposed to do…only when you sit down in the office, then you remember, oh I got to do this…that is all your second priority. Because your ward and your patients come first.” (WN7)

Nevertheless, many understood and observed the benefits that caregivers and their care-recipients reaped through the programme. Indeed nurses like WN7 “[felt] glad [to be] part of Carer Matters” and found it to be “useful” as through the programme, she was able to “understand the carers, what was happening to them in the community, and how she could support them”, while WN4 thought that the “project is good for the older person in the community” as it can help caregivers who “do not know about dementia’ to better ‘manage their emotions and everything” else. WN2 on the other hand, possessed strong positive sentiments about Carer Matters due to the influence of her prior personal caregiving experience. “Because last time, I was taking care of my late grandmother…I felt that if [Carer Matters] was there at that time, it would have helped…because my mum [was] going through some carer stress so personally, I actually support this very much.” (WN2).

The acknowledgement of the programme’s value meant that the nurses and ward champions often perceived Carer Matters as a project that they could leverage on to “do something [beneficial] for [the caregivers]” (WN3), and were more willing to integrate the process of caregiver engagement and recruitment into their daily workflow.

“I understand the aim, the benefit [of Carer Matters], so I really want to help with [recruiting caregivers], see how we can help the older patients and help the family members cope when bringing the patient to home from the hospital.” (WC1)

“…I think [the ward champions and nursing officers] see the value…and they feel [Carer Matters] is a good project. We also hope that in the future, the project itself can be sustained.“ (WN6)

Another interviewee highlighted that it took substantial coordination and support from multiple stakeholders in the hospital to ensure the success of this programme.

“What helps [the adoption of Carer Matter]? I think it is the coordinators [caregiver support nurses] guiding and giving us information on the ground, as well as the [ward] champions and sister [ward nurse manager] who is in charge of us, who constantly remind us.” (WN3)

### Adoption by community partners and facilitators/barriers

As Carer Matters remains in its pilot stage, collaboration with community partners was limited to a few organisations. These community partners offer social services ranging from senior day care services to case management services that may support caregivers in easing the transition of their care-recipients from hospital to home. At present, a few caregivers were referred to each community partner. However, the caregiver support nurses expressed difficulty in collaborating with more community partners as not all have services that provide direct support to caregivers, or see the patient and caregiver as a dyadic beneficiary of care.

“The community partners [need to be] on board this [mindset of the] dyad role [between] care-recipient and caregiver. But I don’t know whether the community partners are ready or have the resources to support this. We need them to have the same aim of supporting the caregiver.” (CS2)

Furthermore, community partners mostly provided services within certain geographical boundaries. Hence, caregivers and care-recipients who do not stay within their boundary would need to seek an alternative provider. However, this service gap can be addressed by gathering more community partners on board Carer Matters to support more caregivers with their care recipients’ transition home from the hospital.

### Implementation

Under “implementation” we highlight key adaptations, specifically the use of digital technologies as a response to the COVID-19 situation and recommend the use of a multimodal engagement strategy to improve programme accessibility.

### Adaptability and digitalisation

Due to the constantly evolving COVID-19 situation, Carer Matters had to adapt to confront unexpected challenges. With COVID-19 restrictions in place, many of the programme’s activities were rapidly digitalized. Examples of digital technologies utilized include posters and collaterals with QR codes, and animated videos to publicise the programme and encourage caregivers to sign up. Such digital platforms facilitated caregiver sign-ups for the programme, even amongst older caregivers.

“So it is kind of easy, it is good, this form. People like me [elderly]…I [also] can go through it. Get it done [complete it].” (CG3) Furthermore, the setting of caregiver training courses was switched from on-site to the online platform Zoom. As a result, digital literacy was identified to be a possible challenge among some of the older caregivers. To pre-empt technical issues, caregivers were taught how to use Zoom with their devices prior to the start of the course. Changes to the courses’ content, such as replacing exercises involving simulated patients with discussion time among facilitators and caregivers, were also made, which some caregivers found more informative as “the speakers were very well-informed in the various topics’ as ‘they had done a lot of research and read-up.” (CG18).

Despite the challenges brought about by digitalization, the convenience of participating in online courses attracted many caregivers. It meant that caregivers no longer needed to seek alternative care arrangements for their care-recipients and travel to the hospital to attend the training. Instead, they could simply log in to attend at a place conducive for them.

“It is not going to be long, it is Zoom…that means I can log in from home, I do not have to spend to (travel).” (CG21)

### Accessibility as a guiding principle

Caregivers who joined Carer Matters shared different needs given their diverse socio-demographic profiles. For example, they expressed different preferences for engagement media — such as hardcopy or softcopy caregiving resources, individualized services or bigger group training courses, or customized courses catered to caregivers whose loved ones are at different stages of their dementia disease, a view reinforced by CG15.

“…when you go for a CARERS program like that…it is a general thing…it covers everything…the discussion is not in line…the scope is very wide…[our care-recipients] are going through different stages of [dementia]…if [the content] was…a bit more specific, it would help.” (CG15)

It is therefore of high importance that as Carer Matters proceeds past its pilot share, to consider these preferences in its design to ensure accessibility of its resources and courses.

### Maintenance

Under “maintenance” we highlight the importance in adopting a multi-pronged approach in recruiting caregivers into Carer Matters and explore the possibility of leveraging on healthcare support staff and experienced caregivers to support other caregivers in their journey.

### Multi-pronged approach in recruitment of caregivers

As Carer Matters is still in its pilot phase, more time and manpower resources are needed to reach more caregivers and support them through their care-recipients’ transition home. Study participants suggested additional means to attract caregivers, such as mobilizing other healthcare providers or support staff like patient service associates to recruit caregivers, thereby reducing reliance on the ward nurses alone. This multi-pronged approach was underscored by a hospital leader who believed that caregivers should be more involved in their enrolment, for the sustainability of Carer Matters:

“So, in access to care, there must be multi-pronged approach. Leveraging on the ward nurses is only one approach and we shouldn’t bank all our efforts on one approach.” (HL1)

“Consider ways in which we can bring people to Carer Matters, and there is a motivating factor behind that. Meaning that, how do you nudge people, how do you first draw attention to this program… advertising, marketing right, they are able to capture your attention within that short span one or two minutes, and it does last. So how can we better work on that.” (HL1)

### More caregiver support nurses/ staff needed

Caregiver support nurses play a vital role in the implementation of Carer Matters. From identifying caregivers at risk of stress, offering timely follow-up and conducting caregiver training courses, caregiver support nurses verbalised the need to have more trained nurses to offer these support to caregivers.

“Because right now there are not many caregivers [during pilot phase], yet we are quite packed with conducting the programs. So, I think if the resources remain to be only three of us, it will not be enough. Because other than that, we still have to conduct tele-consult, which requires follow up. Yeah. So, the thing is, is it sustainable if it’s just us.” (CS2)

Moreover, there are caregivers who may need help from caregiver support nurses after office hours to offer advice during an emergency. In order to provide a 24-hour  hotline such that expert knowledge and availability of referral assistance would always be available to caregivers, there is a need to increase the pool of trained nurses to support the caregivers or to have them train healthcare support staff to assist them.

### Leveraging on the support of experienced caregivers

Similarly, there were also suggestions to sustain the programme through recruiting experienced caregivers onboard the team, to support other caregivers on their caregiving journey, in hopes of building a caregiver network that would eventually produce a self-sustaining caregiver support group.

“There are a few participants who are very activated and more willing to share. So- see whether- are they willing to be part of our team? And support the other caregivers.” (CS1)

## Discussion

We aimed to explore the feasibility of Carer Matters, a holistic caregiver-centric hospital-to-home programme, specifically examining its reach, adoption, effectiveness, implementation, and maintenance through the perspectives of various stakeholders. Generally, the findings demonstrated that Carer Matters was effective in improving caregivers’ knowledge, stress levels and confidence in caring for their loved ones—reflected in both disease knowledge scores and training satisfaction together with positive qualitative feedback detailing how they appreciated the support available through the programme. However, challenges were faced in its implementation. This included the difficulty in reaching out to caregivers who would often prioritize the care of their loved ones over their own well-being. Our study also revealed challenges, some of which were brought on by the COVID-19 pandemic situation, to the initial adoption of Carer Matters by stakeholders, including ward nurses and community partners.

Our findings are in congruence with current literature which suggests that much like healthcare providers, family caregivers tend to prioritize the care of patients, often causing the latter to neglect their own well-being [9, 10, 35]. This could possibly explain the ward nurses’ initial resistance towards adopting the programme as part of their daily workflow, and the caregivers’ reluctance towards accepting formal support. Nevertheless, when ward nurses began to see the potential benefits of the programme towards both caregivers and their care-recipients, they were more willing to go the extra mile in engaging caregivers and introducing the programme to them.

Caregivers themselves also realized these benefits, having gained greater awareness on the importance of caring for themselves after receiving caregiving resources and support from Carer Matters. In fact, many caregivers expressed that the tele-support and training courses were instrumental in providing them with emotional support, improving their coping ability, and reducing their caregiver burden and their sense of isolation.

Admittedly, social-distancing measures imposed during the COVID-19 pandemic had brought about additional implementation challenges and might have affected the effectiveness of the training courses. However, the team was able to learn and adapt quickly to the volatile environment by leveraging on technology to deliver resources and courses online. In the process, participating caregivers were trained to utilize the online platform Zoom, and were able to attend and benefit from the courses in the comfort of their homes.

The study findings reiterated the need to advocate for a patient-caregiver dyadic hospital-to-home framework [11, 36]. From a systems perspective, it is crucial to improve the integration of this care framework into existing care structures in two ways.

Firstly, Carer Matters’ stakeholders — including healthcare providers, and caregivers themselves —tend to focus on the patient-centred mindset of care provision, which may overlook the well-being of family caregivers. In congruence with Carer Matters, Adelman et al. [17] underscored the need for healthcare providers to recognize that the well-being of family caregivers is equally important to that of their patients, and the quality of care provided at home. Hence, the shift in perspective towards the patient and caregiver as a unitary beneficiary of institutional support is imperative in establishing and sustaining a holistic model of care such as Carer Matters. As evident from our study findings, the engagement of relevant stakeholders on different levels is crucial in facilitating such hospital-wide change. In particular, our findings highlighted the vital role that ward champions played in encouraging their fellow ward nurses to engage with caregivers. Also, findings revealed that the recognition and endorsement by hospital leaders enabled Carer Matters to garner the support of other healthcare providers in the hospital with greater ease. This aligned with observations from Greenhalgh et al. [37] that the distribution of appointed leaders among individuals and leading organisations is crucial to sustain such a change.

Secondly, Carer Matters demonstrated the value of extending the patient-focused hospital-to-home framework to include caregivers. The approach of holistic caregiver support was appreciated and valued by caregivers, nurses, clinicians, and other healthcare providers. This complements the current hospital-to-home care delivery for patients, aimed at helping them thrive with a wellspring of support at home and beyond [11]. In the current system, typically only the very stressed caregivers who are in crisis and those with complex financial situations, are referred to the medical social workers. Our programme compliments current care by establishing a framework that includes structured screening and assessment of caregivers so that proactive care for the caregivers could be provided to equip them for both their current and future caregiving needs.

The COVID-19 crisis has led to an unprecedented shift to the digitalization of health care where care and services are now more frequently provided through telecommunication technologies [38]. With restrictions on hospital visitations and increased workload among ward nurses, Carer Matters has pivoted towards digital technology to recruit, educate, equip and support caregivers. Arguably, the shift towards digitalization may pose challenges to recruit caregivers of lower digital literacy, especially older caregivers. However, national efforts are being made in Singapore to improve digital literacy among older adults, easing their entry into using video-communication tools and smart devices as part of everyday life [39]. Moving forward, the programme will adopt a hybrid approach which not only leverages on technology for online courses and tele-support, but also offers in-person training courses whenever possible. The use of both high-tech and low-tech strategies ensure a balance between digital accessibility and personal touch in delivering helpful and timely information to family caregivers [40]. Given the busy ward environment, the programme will also explore multi-pronged method to enrolled caregivers into the programme, including self-referral, instead of relying on nurses alone. We will also explore the possibility of training and upskilling healthcare support staff to assist in the training and in the care of the caregivers.

### Strengths and limitations

This study is one of the first to explore the feasibility of a holistic hospital-to-home programme, that focuses on the equipping caregivers to care for both their patients and themselves. Our study offered insights into the challenges of implementing such a structured programme, especially during a pandemic. The strengths of this study include its sampling strategy to ensure heterogeneity. A range of stakeholders from both the hospital and community settings, and including both healthcare users and providers, were recruited to provide multiple perspectives on their perceptions and experiences with Carer Matters and its feasibility. Moreover, qualitative and quantitative data were triangulated to provide a holistic perspective of the programme’s effect on both caregivers and the healthcare ecosystem.

 COVID-19 pandemic visitation restrictions limited the enrolment of caregivers into our programme and our training courses over the pilot period. Our model of receiving sign-ups through the electronic needs assessment, while novel, prevented us from better understanding the sampling frame, overall response rate, and the characteristics of non-participants, as it did not capture information on non-participating caregivers. Furthermore, caregivers of minority ethnicities such as the Malays and Indians could have been better represented in our study. Future studies can investigate the experiences of those caregivers to provide greater insights. Future studies are also needed to examine the long-term effects of caregiver-centric support on patient-specific outcomes such as changes in hospital length of stay or readmission and institutionalization rates.

## Conclusions

This feasibility study highlights that a holistic, hospital-to-home framework is a valuable complement to the ecosystem of support for caregivers and their care recipients. This framework ensures that caregivers are sufficiently equipped with the skills and knowledge to provide care without neglecting their own well-being. In order to sustain such a framework, we believe that healthcare systems should embrace the caregiver-patient dyad as a unitary beneficiary of care, yet we also recognize that such a shift in mindset requires time. Despite the challenges faced during the pilot implementation of Carer Matters, its chief objective of rendering adequate and timely support to caregivers was achieved. Moreover, given that majority of the challenges faced were brought about by the COVID-19 pandemic, we anticipate that these challenges would diminish with the move towards an endemic environment. Coupled with the paradigm shift in mindset, Carer Matters’ holistic support of caregivers can improve the sustainability of “ageing in place” as a solution for ageing populations living in the community.

## Supplementary Information

**Additional file 1.**

**Additional file 2.**

## Data Availability

The datasets generated and/or analysed during the current study are not publicly available as the ethical approval for the study does not permit data sharing, but are available from the corresponding author on reasonable request.

## Abbreviations

RE-AIM: The Reach, Effectiveness, Adoption, Implementation, and Maintenance framework
CARERS: The Coaching, Advocacy, Respite, Education, Relationship, Simulation programme
