# Supplementary material for: Applying the RE-AIM framework to evaluate a holistic caregiver-centric hospital-to-home programme: a feasibility study on Carer Matters
Source: BMC Health Serv Res. 2022 Jul 19;22:933. doi: 10.1186/s12913-022-08317-3 (PMC9296119; doi:10.1186/s12913-022-08317-3)
Supplement: Supplementary file 1 — Additional file 1. [file 12913_2022_8317_MOESM1_ESM.pdf]

Carer Matters Pilot Study Interview Guide

Family Caregiver: (Questions asked according to the component of Carer Matters joined)

Reach

How do you know about Carer Matters? (Prompt: Who introduced Carer Matters to you?)

What makes you want to complete the assessment form/survey?

How did you find the process of completing the assessment form/survey? Did you encounter any difficulties in the process? If yes, what are they?

How can we improve on this process? (How can we better reach caregivers? How can we better encourage caregivers to complete the assessment form?)

Implementation

What encourages you to continue with Carer Matters programme activities?

How can we continue to engage/ reach out to caregivers after the assessment form?

Teleconsult: Do you remember when you received the first phone call from the caregiver support nurses (to find out how you are doing)?

Teleconsult: Do you remember what was discussed during these call(s)?

Caregiver training programmes: Which caregiver training programme did you participate in? How did you find the programme?

Caregiver training programmes: Do share with me your experience with the <programme>. (How was <the programme> being conducted? By whom and when was it conducted? Was it engaging?)

Effectiveness

Tele-support: What do you find valuable through the follow up calls?

Tele-support: Would you want to continue to receive follow up calls? If yes, why? If not, why?

Tele-support: How can we improve/ do better in supporting caregivers through follow-up calls?

Caregiver training programmes: In what way was <the programme> useful or not useful? (Prompt: What have you gained from <the programme>? Was the programme able to address your needs as a caregiver for your care recipient? If yes, in what ways? If not, why not? Have the sharing by the other caregivers in the program been useful? Yes/no. Why?)

Caregiver training programmes: What do you think made the <programme> successful? What made it difficult for you to learn through the program?

Caregiver training programmes: How do you think the programme can be improved?

Caregiver training programmes: Will you recommend the caregiver programme to other caregivers? Why?

Reach

How can we better reach caregivers or encourage them to join the programs?

Maintenance

How can we continue to engage/ reach out to caregivers post programs?

Ward nurses/Ward Champions

[Involved in Intervention 1 (Identify) and Intervention 2 (Screening + Needs Assessment)]

<u>Adoption</u>

What is your role in the Carer Matters as a ward nurse/ward resource nurse? How long have you been in this role with Carer Matters?

What are your experiences in approaching the family caregivers of older adults? (Were the family caregivers receptive? Was it difficult to approach them?)

What are the challenges you faced in approaching/ screening the family caregivers. (Why do you think the family caregivers were willing or unwilling to complete the needs assessment form?)

How have you tried to overcome these challenges?

<Show the proposed TOC> We have developed a theory of change to depict what a typical caregiver would go through with Carer Matters. We have mapped out the key activities of Carer Matters, and how they would contribute to the outcomes.

<u>Effectiveness</u>

What are your views on the intended outcomes of the programme?

Do you think the outcomes we have developed are within the reach of the programme?

What other outcomes would you envision the programme to achieve? (if any)

<u>Implementation</u>

What are your views about the activities of the programme? What other activities can we include into the programme to achieve its outcomes?

Why do you think some caregivers do not participate / sign up for the caregiver programs? Why do you think others participate in the programme?

Which group of caregivers do you think are most likely to participate?

<u>Adoption</u>

How do you find the workflow of the screening process? How do you think the current screening workflow can be improved?

How do you find the caregiver screening tool? Which group of caregivers do you think benefits most from the caregiver screening tool?

What do you think can be done to encourage ward nurses to actively screen and refer family caregivers to Carer Matters?

<u>Maintenance</u>

How can we continue to engage/reach out to caregivers?

Caregiver support nurses

[Involved in all Interventions]

1) **Identify, 2) Screening + Needs Assessment, 3a) Offer Tele-support, 3b) Conduct Caregiver Training Programmes, 4) Engage Community Partners, 5) Establish Caregiver Support Network**

<u>Adoption</u>

What is your role in the Carer Matters as a caregiver support nurse? How long have you been working with Carer Matters?

How do you work with the ward champion/ward nurses for Carer Matters? How would you describe your working relationship?

What do you think are the barriers that prevent ward nurses from referring the caregivers to Carer Matters

<u>Reach</u>

Why do you think the family caregivers are willing/unwilling in completing the needs assessment form?)

Based on your experience working with family caregivers, which group(s) of caregivers will the screening tools and needs assessment form be the most useful to/helpful for?

Tele-support: What are your experiences in conducting tele-consult with family caregivers? (Were the caregivers receptive in receiving follow-up calls?)

<u>Implementation</u>

Tele-support: How was the tele-support being conducted? When do you conduct the tele-support?

How often do you follow up with the caregivers through tele-support?

<u>Effectiveness</u>

How did the caregivers react when you first call them?

How do you think the tele-support benefits the caregivers?

What are the factors that led to successful or unsuccessful follow-up calls?

<u>Implementation</u>

Caregiver programs: Can you name me the different caregiver programmes offered to family caregivers? How do you decide who (type of caregivers) to offer these programmes to? How often were the different caregiver programmes being conducted?

What do you think contribute to the uptake of these caregiver programmes?

What are the challenges you faced in conducting the caregiver programmes?

What have you done to overcome these challenges?

TOC:

<Show the proposed TOC> We have developed a theory of change to depict what a typical caregiver would go through with Carer Matters. We have mapped out the key activities of the programme, and how they would contribute to the outcomes.

Effectiveness

What are your views on the intended outcomes of the programme?

Do you think the outcomes we have developed are within the reach of the programme?

What other outcomes would you envision the programme to achieve? (if any)

What do you think facilitates the achievement of these intended outcomes?

What do you think impedes the achievement of these intended outcomes?

Implementation

What are your views about the activities of the programme? What other activities can we include into the programme to achieve its outcomes?

Why do you think some caregivers do not participate / sign up for the caregiver programs? Why do you think others participate in the programme?

What other challenges do you face as a caregiver support nurse? How do you think you can be better supported as a caregiver support nurse?

Hospital leaders:

[Interviewed based on all 5 interventions]

Introduction:

Can you share with me on your role as a leader in the hospital? Can you share how much you know about Carer Matters?

<Show the proposed TOC> We have developed a theory of change to depict what a typical caregiver would go through with Carer Matters.

Effectiveness

What are your views on the intended outcomes of the programme?

Do you think the outcomes we have developed are within the reach of the programme?

What other outcomes would you envision the programme to achieve? (if any)

What are your views about the activities of the programme? What other activities do you think we can include into the programme to achieve its outcomes?

REACH

Why do you think family caregivers participate/ do not participate in Carer Matters? How do you think we can improve to better reach out to the caregivers?

Adoption

What do you think encourages ward nurses to actively refer caregivers to Carer Matters? What do you think are the barriers that prevent them from reaching out to the caregivers?

Maintenance

How do you think the Carer Matters align with nursing/the organisational direction?

What are the challenges you foresee in sustaining this program such that it continues to benefit family caregivers in the long run? (and eventually the community and the hospital)

Community based clinicians/ Community partners[C1-5]:

[Involved in Intervention 4 as community partners]

<u>Introduction:</u>

Do you know about Carer Matters? Can you share with me how much you know about the programme? What is your role with Carer Matters?

<Show the proposed TOC> We have developed a theory of change to depict what a typical caregiver would go through with Carer Matters.

<u>Effectiveness</u>

What are your views on the intended outcomes of the programme?

Do you think the outcomes we have developed are within the reach of the programme?

What other outcomes would you envision the programme to achieve? (if any)

What are your views about the activities of the programme? What other activities do you think we can include into the programme to achieve its outcomes?

<u>REACH</u>

Why do you think family caregivers participate/ do not participate in the programme? How do you think we can improve to better reach out to the caregivers?

<u>Implementation</u>

How do you, in your capacity as <MSW/ OT/PT/ Doctor> play a role in Carer Matters?

As this program is still in its pilot phase, what challenges do you foresee in making this programme available to other wards and subsequently the entire hospital?

<u>Maintenance</u>

How do you think the Carer Matters can complement your work as a MSW/ PT/ OT/ Doctor?

What are the challenges you foresee in sustaining this program such that it continues to benefit family caregivers in the long run?
