# Supplementary material for: Applying the RE-AIM framework to evaluate a holistic caregiver-centric hospital-to-home programme: a feasibility study on Carer Matters
Source: BMC Health Serv Res. 2022 Jul 19;22:933. doi: 10.1186/s12913-022-08317-3 (PMC9296119; doi:10.1186/s12913-022-08317-3)
Supplement: Supplementary file 2 — Additional file 2. [file 12913_2022_8317_MOESM2_ESM.pdf]

Caregiving Activities

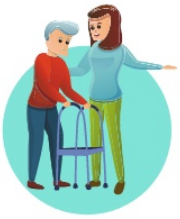

Basic activities of daily living:

Feeding, Bathing, Walking

Shopping, Housekeeping, Food preparation

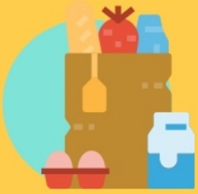

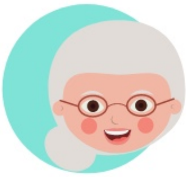

Ensuring physical and mental well-being

Managing medical condition(s)

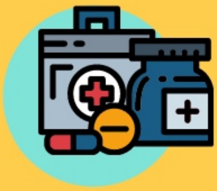

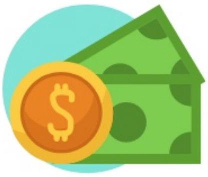

Managing finances

Decision making

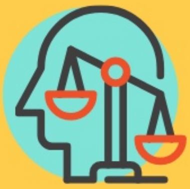

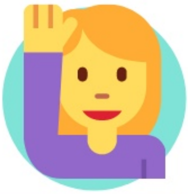

Advocacy needs

Coordination of care needs

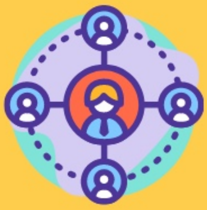

Reference: Committee on Family Caregiving for Older Adults; Board on Health Care Services; Health and Medicine Division; National Academies of Sciences, Engineering, and Medicine. (2016). Families Caring for an Aging America: Family Caregiving Roles and Impacts (R. Schulz & J. Eden J, Ed.). National Academies Press (US).

© Project Carer Matters

Physical Impacts of Caregiving

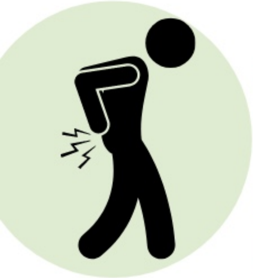

Back pain and muscle aches

Resulting from poor transferring techniques, improper posture or lack of assistance

Weight loss or gain

From changes in eating habits

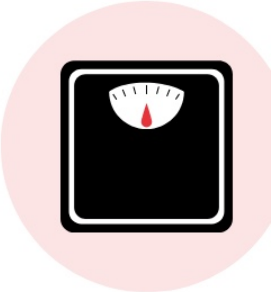

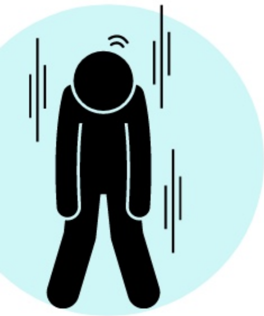

Fatigue

From having to juggle multiple roles

Poor sleep

From interrupted rest

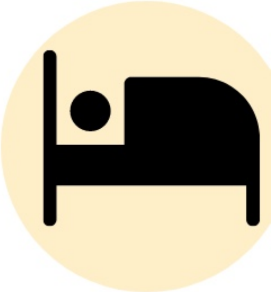

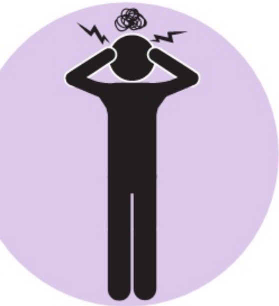

Headache

From insufficient rest

The lack of time and energy may cause you to neglect your own health. It is important to look after yourself while taking care of your care recipient.

Practise self-care

Reference: Committee on Family Caregiving for Older Adults; Board on Health Care Services; Health and Medicine Division; National Academies of Sciences, Engineering, and Medicine. (2016). Families Caring for an Aging America: Family Caregiving Roles and Impacts (R. Schulz & J. Eden J, Ed.). National Academies Press (US).

© Project Carer Matters

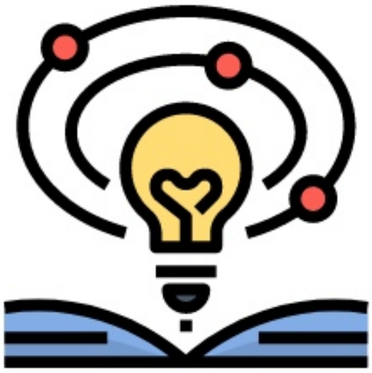

Knowledge and Skills of Caregiving

What caregiver knowledge and skills do I need?
Where can I find them?

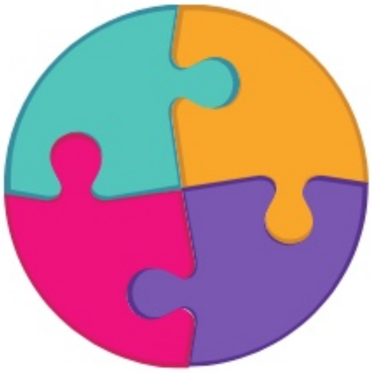

Strategies to Cope with Caregiving

How can I cope with the challenges and demanding nature of caregiving?

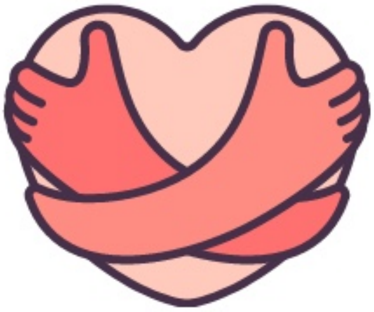

Supporting Yourself

How can I care for myself?

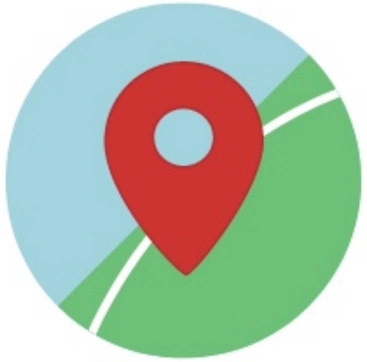

System Navigation

What are the available community resources?
Where can I find them?

© Project Carer Matters

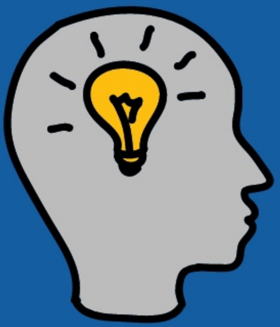

Problem-solving Technique

5 steps to help you problem solve during your caregiving journey.

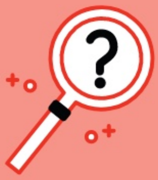

1. Define the Problem

Identify the root problem

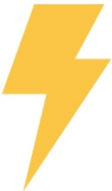

2. Brainstorm Solutions

Think of all possible solutions and write them down

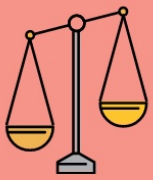

3. Weigh Pros & Cons

List the benefits and drawbacks of each solution

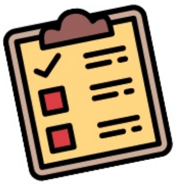

4. Choose Solution and Action Plan

- Choose the solution with the most benefits and least drawbacks
- Plan out how you will carry out the solution

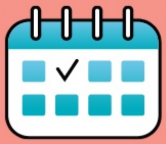

5. Evaluate Outcomes

Set a timeline to check if the solution is working

Reference: Cleveland Clinic. (2018, August 20). Caregivers: Successful Caregiving. Clevelandclinic. https://my.clevelandclinic.org/health/articles/9226-caregivers-successful-caregiving

© Project Carer Matters
